# Effectiveness and acceptance of Vestibulo-Ocular Reflex adaptation training in children with recurrent vertigo with unilateral vestibular dysfunction and normal balance function

**DOI:** 10.3389/fneur.2022.996715

**Published:** 2022-12-16

**Authors:** Ning Ma, Handi Liu, Bing Liu, Li Zhang, Bei Li, Yang Yang, Wei Liu, Min Chen, Jianbo Shao, Xiao Zhang, Xin Ni, Jie Zhang

**Affiliations:** ^1^Department of Otorhinolaryngology Head and Neck Surgery, Beijing Children's Hospital, Capital Medical University, National Center for Children's Health, Beijing, China; ^2^Beijing Key Laboratory for Pediatric Diseases of Otolaryngology Head and Neck Surgery, Beijing, China

**Keywords:** vestibular rehabilitation, recurrent vertigo of childhood, unilateral vestibular dysfunction, children, Vestibulo-Ocular Reflex

## Abstract

**Objective:**

This was a block randomized controlled study to evaluate the effectiveness and acceptance of Vestibulo-Ocular Reflex (VOR) adaptation training in children with recurrent vertigo with unilateral vestibular dysfunction (UVD) and normal balance function.

**Methods:**

Thirty children, aged 4–13 years, diagnosed with recurrent vertigo of childhood (RVC) with UVD (according to a caloric test) and normal balance function were analyzed. These 30 children were divided into 10 blocks based on similar age and severity of vertigo. Three children in each block were randomly assigned to one of three groups to receive 1 month of treatment. Group A received vestibular-ocular reflex (VOR) adaptation training, Group B received Cawthorne-Cooksey training, and a control group received no training. All children were administered pharmacotherapy [Ginkgo biloba leaf extract (drops)]. The Dizziness Handicap Inventory (DHI), Visual Analog Scale of Quality of Life with Vertigo (VAS-QLV), and canal paralysis (CP) on the caloric test were recorded before and after treatment, and the effectiveness of treatment was evaluated. The Visual Analog Scale of Acceptance (VAS-A) was used to evaluate the acceptance of the training in the two groups that received training.

**Results:**

There were 10 children each in Group A, Group B, and the control group; the male to female ratio was 1, and the average age in each group was 9.0 ± 3.2, 8.4 ± 3.0, and 8.3 ± 2.6 years, respectively. The effective rate was 100% in Group A, 65% in Group B, and 60% in Group C. The recovery rate on caloric testing after treatment was 100, 70, and 50%, respectively. DHI scores before and after training were 56.8 ± 12.4 and 8.8 ± 6.1 in Group A, 57.8 ± 12.6 and 18.8 ± 9.7 in Group B, and 56.8 ± 12.4 and 24.0 ± 15.3 in Group C (all *P* = 0.000). VAS-QLV scores before and after training were 7.5 ± 1.0 and 0.9 ± 0.9 in Group A, 6.4 ± 2.2 and 2.7 ± 1.1 in Group B, and 6.6 ± 1.6 and 2.6 ± 1.4 in Group C (all *P* < 0.05). The CP values before and after training were 35.7 ± 15.1 and 12.9 ± 8.7 in Group A, 33.6 ± 20.1 and 23.6 ± 19.3 in Group B, and 38.6 ± 21.1 and 24.8 ± 17.9 in Group C (*P* = 0.001, *P* = 0.015, and *P* = 0.050, respectively). Between-group comparisons showed that the decreases in DHI and VAS-QLV scores after training were significantly different (*P* = 0.015, *P* = 0.02), while CP values were not (*P* = 0.139). After training, the DHI value had decreased significantly more in Group A compared with Group C (*P* < 0.05), but there were no other differences. After training, VAS-QLV scores in Group A had decreased significantly more compared with Group B and C (*P* < 0.05). In terms of acceptance, the VAS-A score was 7.6 ± 2.2 in Group A and 3.1 ± 2.8 in Group B (*P* =0.004), The acceptance rate was 70% in group A and 10% in group B. there was no significant correlation between age and VAS-A in either group A or group B (*P* > 0.05).

**Conclusion:**

This study strongly suggests that vestibular rehabilitation training should be performed in children with vertigo to improve symptoms. For children with RVC with UVD but normal balance function, a single VOR adaptation program can effectively improve vertigo symptoms, and given its simplicity, time-effectiveness, and excellent outcomes, it is associated with better acceptance in children compared to classic Cawthorne-Cooksey training.

## 1. Introduction

Recurrent vertigo of childhood (RVC) is clearly defined in the clinical guidelines of the Bárány Society and the Vestibular Disorders Classification Committee of the International Headache Society in 2021 (1). Diagnostic criteria of Recurrent Vertigo of Childhood (RVC) include: A: At least three episodes with vestibular symptoms of moderate or severe intensity, lasting between 1 min and 72 h. B. None of the criteria for Vestibular Migraine of Childhood or Probable Vestibular Migraine of Childhood. C. Age < 18 years D. Not better accounted for by another headache disorder, vestibular disorder, or other condition. As this type of vertigo is moderate to severe and occurs repeatedly, it can affect daily life and learning behavior (2), produce anxiety (3), and even affect the child's family. The targeted treatment is vestibular rehabilitation (VR) (4), but VR is not widely used in the clinic. One key issue is the uncertainty of the effectiveness, applicability, and acceptance of VR in children with vertigo. Moreover, there is a lack of corresponding research data on how to improve the effectiveness of treatment in children with RVC, enhance acceptance, and achieve stable short-and long-term effects.

VR includes gaze stability exercises, balance and gait training, and walking for endurance. Gaze stability exercises include adaptation training, alternative training, and habitual training (4). Different exercise approaches have been proposed to address these different problems. Vestibulo-Ocular Reflex (VOR) adaptation training are based on inducing changes in the neuronal response of the vestibular system to a specific error signal retinal slip. The goals of these exercises are to decrease visual blurring during head movement, improve postural stability, and decrease symptoms (5). Most vestibular rehabilitation programs include balance training, gait exercises and endurance training (5). The classic VR is the Cawthorne-Cooksey training (6), which is complex and time taking. Although it can be used in children, persistence, understanding, and compliance are poor, and therefore, training often stops early and/or does not play much of a therapeutic role (7, 8). In our clinical work, we found that for children, increased training time will reduce the children's training compliance and adherence. It leads to the premature termination of training and has a negative impact on the therapeutic effect. A considerable number of children with RVC have unilateral vestibular dysfunction (UVD), but have not shown a balance disorder. For these children, we adopted a targeted and single intensive Vestibulo-Ocular Reflex (VOR) adaptation training only. Reduce the training time and complexity, so as to improve the compliance and completion of vestibular rehabilitation training. In this study, by comparing with the classical Cawthorne-Cooksey training, we investigated the effectiveness and suitability of this simplified VR in children with RVC with a view to improving the therapeutic effect in this patient group.

## 2. Materials and methods

Children aged 4–13 years who visited the outpatient clinic of the Department of Otorhinolaryngology Head and Neck Surgery of Beijing Children's Hospital affiliated to Capital Medical University between 2021 and 2022 with recurrent dizziness as the main complaint were studied. A total of 30 children who met the diagnostic criteria (1) for RVC proposed by the Bárány Society International Classification of Vestibular Disorders (ICVD) and the Vestibular Disorders Classification Committee of the International Headache Society with unilateral peripheral vestibular dysfunction and normal balance function were recruited.

Peripheral vestibular dysfunction was determined by a saccade test, smooth pursuit test, optokinetic test, gaze test, spontaneous nystagmus, caloric test, and positioning nystagmus (Dix-Hallpike test and supine roll test). Saccade tests and gaze tests were normal in all the children. Smooth pursuit tests response type I or type II. Optokinetic tests response: 20 and 40°/s symmetry. There were no spontaneous nystagmus or irrigation nystagmus. Dix-Hallpike tests and supine roll tests were negative. The caloric tests indicate that unilateral horizontal semicircular canal function were hypofunction (9, 10). Head MRI indicated unilateral external semicircular canal dysfunction (11) in one patient. The evaluation of balance function includes the collection of history and the examination of balance function. During the consultation, children said that he could stand and walk normally during the attack of vertigo, without unstable feelings such as stepping on cotton and floating, falling history, and having physical or motor coordination problems. Balance function was examined using the Tandem Romberg test (Mann test), Fukuda stepping test, and past pointing test. Tandem Romberg test (Mann test): Maintain a stable position for more than 10 s. Fukuda stepping test: *in-situ* offset angle < 30°, self-rotation angle < 90°, moving distance < 50 cm. Past pointing test: no finger crossing. Children who meet the above conditions are considered to have normal balance function (12).

The vestibular rehabilitation training programs implemented were the VOR adaptation training and the Cawthorne-Cooksey training, and a control group was set up. All children were administered drugs (Ginkgo biloba extract drops agent) for symptomatic treatment. According to a block random design, children were divided into 10 blocks according to age and vertigo severity. Three children in each block were randomly assigned to each of the three groups (A: the VOR adaptation training group; B: Cawthorne-Cooksey training group C: the control group no training) by lottery, and the course of treatment in each group was 1 month. Informed consent was signed by the guardian of all children. This study was approved by the Hospital's Ethics Committee.

Inclusion criteria included: (1) diagnosis of RVC (at least three episodes with vestibular symptoms of moderate or severe intensity, lasting between 1 min and 72 h).All the children enrolled had recurrent vertigo episodes in the past 1–2 months at that time, ranging from once a day to several times a day; (2) unilateral peripheral vestibular dysfunction; (3) normal balance function; (4) bilateral ear canal patency and tympanic membrane integrity examined by otoscopy; (5) normal hearing thresholds (children 6 years and under) underwent pediatric behavioral audiometry using Interacoustics AD229b (Interacoustics; Middelfart, Denmark) and children over 6 years underwent pure tone audiometry using Conera (GN Otometrics; Copenhagen, Denmark); (6) type A tympanogram.

Exclusion criteria included: (1) ophthalmologic and central vertigo confirmed by ophthalmology and neurology; (2) benign paroxysmal positional vertigo; (3) vestibular migraine (1); and (4) possible vestibular migraine (1).

### 2.1. Evaluation methods

#### 2.1.1. Vestibular caloric test

The canal paralysis (CP) value was calculated and recorded. CP was considered abnormal if the CP value was more than 25% (9, 10, 13).

#### 2.1.2. Dizziness Handicap Inventory (DHI)

A total of 25 questions were asked, and the answer options were: four points for yes, two points for sometimes, and 0 points for no. A score of 0–30 indicated a mild disorder, 31–60 indicated a moderate disorder, and 61–100 indicated a severe disorder (14). Reduction of the DHI was evaluated.

In order to control the quality of the questionnaire, all the questions were given to the children after they were interpreted by the vestibular studio technician and parents.

#### 2.1.3. Visual Analog Scale-Quality of Life with Vertigo (VAS-QLV)

A total of two questions were asked: (1) the impact of vertigo on daily life and (2) the impact of vertigo on learning behavior. Possible ratings were 0–10 points, with 0 points indicating no effect and 10 points indicating complete inability to partake in normal activity and learning behavior. The two questions were scored separately, and the average score was taken. A score of 0–3 indicated a mild effect, 4–6 indicated a moderate effect, and 7–10 indicated a severe effect.

#### 2.1.4. Acceptance

The Visual Analog Scale-Acceptance (VAS-A) score was calculated from a total of two questions that covered: (1) understanding of the vestibular rehabilitation training program and (2) completion of the vestibular training program. Possible ratings were 0–10 points, with 0 points indicating no understanding or no training and 10 points indicating full understanding or complete training according to the plan and frequency. The two questions were scored separately, and the average score was taken. A score of 0–3 points indicated poor acceptance, 4–6 points indicated general acceptance, and 7–10 points indicated good acceptance. The acceptance rate and the completion rate were assessed after treatment in both groups. The acceptance rate was the number of good acceptability results measured by VAS-A/10.

### 2.2. Training method

#### 2.2.1. Group A (VOR adaptation training group)

Training included shaking head fixation, alternate fixation, separation fixation, and reverse fixation for a total of 5 min (15). The specific methods were as follows: (1) shaking head fixation: fix the eyes on the target in front and turn the head from left to right and up and down; (2) alternate fixation: the eyes look at the target objects up, down, left, and right, and the head moves with the eyes at the same time; (3) separation fixation: the eyes look at the target objects up, down, left, and right, and the eyes move first, the head moves later; and (4) reverse fixation: the eyes look at the target objects up, down, left, and right, and the head moves in the opposite direction as the eyes. Five repetitions of each movement were completed in every training session.

#### 2.2.2. Group B (Cawthorne-Cooksey training group)

Cawthorne-Cooksey exercises (6) represent a general approach to vestibular rehabilitation and include a standardized series of exercises that involve a progression of eye movements only, head movements with eyes open or closed, bending over, sitting-standing, tossing a ball, and walking.

#### 2.2.3. Group C (control group)

This group received no training.

We made videos to guide children to follow the training programs, including the explanation of movements and suggestions on the angle and speed of turning the head. Group A received 5 min of video training 3 times a day for 1 month. Group B received 17 min of video training 3 times a day for 1 month. The children were supervised by their parents to watch the video and train at home, and the training was recorded daily. The patients were followed up by telephone every week, followed up by a follow-up visit at the end of the 1-month course of treatment.

### 2.3. Efficacy evaluation

The caloric test was conducted before and after treatment to compare the recovery of vestibular function and evaluate the decline of CP value. The DHI and the VAS-QLV were completed with the assistance of the child's guardian. The evaluation method was as follows: the efficacy was evaluated according to the difference of DHI score and VAS-QLV before and after training. The improvement of moderate and severe disorder to mild disorder was considered effective. The VAS-A score was used to evaluate the acceptance of the training programs in Group A and Group B. The acceptance rate and the completion rate were assessed after treatment in both groups.

### 2.4. Statistical analysis

SPSS Statistics 21 software was used for statistical analysis according to a block randomized controlled design. For CP values, DHI scores, and VAS-QLV scores, two-way ANOVA was used between the overall groups, different time points and the interaction between time and group. ANOVA was used for the overall comparison between the three groups at the same time point, LSD method was used for the comparison between the two groups, and paired *t*-test was used for the before and after comparison of the same group. Pearson correlation analysis was used between age and VAS-A correlation analysis. Statistical significance was set at *P* < 0.05.

## 3. Results

There were 10 children in group A, group B and group C, the male to female ratio was 1:1:1, the minimum age was 4 years, the maximum age was 13 years, the average age of each group was 9.0 ± 3.2, 8.4 ± 3.0, 8.3 ± 2.6, respectively.

The frequency of attacks in all patients ranged from 2 to 3 times per week to 7 to 8 times per day in the month before their visit. The duration of the chief complaint of all the children was counted. The duration of the chief complaint was defined as the duration from the first onset of vertigo to the time of diagnosis. Patients can be classified as having a visit within 2 weeks, 3 months, or more than 3 months. The results are shown in the Table 1. The number of DHI and VAS-QLV severity of each group before and after treatment are shown in Table 2. Group A showed 100% efficiency, group B showed 65% efficiency, and group C showed 60% efficiency. The recovery rate on the caloric tests after treatment was 100, 70, and 50% (*P* = 0.350), respectively.

**Table 1:** Duration of chief complaint.

	**N1**	**N2**	**N3**
Group A	3	4	3
Group B	4	3	3
Group C	4	3	3

The duration of the chief complaint: the duration from the first onset of vertigo to the time of diagnosis. N1, the number of children who complained within 2 weeks; N2, the number of children who complained within 3 months; N3, the number of children who complained over 3 months.

**Table 2:** The scores of DHI and VAS-QLV severity before and after treatment.

		**Group A (** ***n*** **)**		**Group B (** ***n*** **)**		**Group C (** ***n*** **)**	
		**Pre-treatment**	**After-treatment**	**Pre-treatment**	**After-treatment**	**Pre-treatment**	**After-treatment**
DHI	Mild	0	10	0	7	0	6
	Moderate	7	0	7	3	7	4
	Severe	3	0	3	0	3	0
VAS-QLV	Mild	0	10	0	6	0	6
	Moderate	2	0	2	4	2	4
	Severe	8	0	8	0	8	0

The results of CP are shown in Tables 3, 4.

**Table 3:** Results of two-way ANOVA of CP.

**Source**	**Type III sum of squares**	**df**	**Mean square**	***F***	***P***	**η**
Group	552.400	2.000	276.200	1.044	0.359	0.037
time	6,000.001	1.000	6,000.001	22.683	< 0.001	0.296
Group*time	289.200	2.000	144.600	0.547	0.582	0.020

**Table 4:** Comparison of CP between and within groups before and after treatment.

**Group**	**CP-pre**	**CP-post**	**CP-difference**	**Paired *t*-test**	***P***
A	35.7 ± 15.1	12.9 ± 8.1	22.8 ± 15.2	4.752	0.001
B	40.3 ± 19.9	16.9 ± 11.4	23.4 ± 24.7	2.995	0.015
C	38.6 ± 21.1	24.8 ± 17.9	13.8 ± 19.3	2.264	0.050
F	0.152	2.129	0.716		
P	0.860	0.139	0.498		

The results of two-way ANOVA showed that there was no statistically significant difference between the overall groups (*P* > 0.05), there was a statistically significant difference between different time points (*P* < 0.05), and the interaction between time and group was not statistically significant (*P* > 0.05).

The comparison of treatment effect showed that CP values did not have a statistically significant difference (*P* = 0.498). The before and after CP values were 35.7 ± 15.1 and 12.9 ± 8.1 in Group A, 33.6 ± 20.1 and 23.6 ± 19.3 in Group B, and 38.6 ± 21.1 and 24.8 ± 17.9 in Group C (*P* = 0.001, *P* = 0.015, and *P* = 0.050, respectively).

The results of DHI are shown in Tables 5, 6.

**Table 5:** Results of two-way ANOVA of DHI.

**Source**	**Type III sum of squares**	**df**	**Mean square**	***F***	***P***	**η**
Group	616.133	2.000	308.067	2.221	0.118	0.076
time	23,920.067	1.000	23,920.067	172.454	< 0.001	0.762
Group*time	584.133	2.000	292.067	2.106	0.132	0.072

**Table 6:** Comparison of DHI between and within groups before and after treatment.

**Group**	**DHI-pre**	**DHI-post**	**DHI-difference**	**Paired *t*-test**	***P***
A	56.8 ± 12.4	8.8 ± 6.1^*^	48.0 ± 9.3^*^	16.347	< 0.001
B	57.8 ± 12.6	18.8 ± 9.7	39.0 ± 10.4	11.831	< 0.001
C	56.8 ± 12.4	24.0 ± 15.3	32.8 ± 13.3	7.814	< 0.001
F	0.021	4.896	4.723		
P	0.979	0.015	0.017		

* Indicates a statistically significant difference from group C (*P* < 0.05).

The results of two-way ANOVA showed that there was no statistically significant difference between the overall groups (*P* > 0.05), there was a statistically significant difference between different time points (*P* < 0.05), and the interaction between time and group was not statistically significant (*P* > 0.05).

The before and after treatment scores on the DHI were 56.8 ± 12.4 and 8.8 ± 6.1 in Group A, 57.8 ± 12.6 and 18.8 ± 9.7 in Group B, and 56.8 ± 12.4 and 24.0 ± 15.3 in Group C, demonstrating a significant improvement in all groups (all *P* < 0.001). The comparison of treatment effect showed that DHI scores differed significantly between groups (*P* = 0.017). After training, the DHI in Group A decreased significantly compared with Group C (*P* < 0.05).

The results of VAS-QLV are shown in Tables 7, 8.

**Table 7:** Results of two-way ANOVA of VAS-QLV.

**Source**	**Type III sum of squares**	**df**	**Mean square**	***F***	***P***	**η**
Group	1.900	2.000	0.950	0.481	0.621	0.017
time	340.817	1.000	340.817	172.485	< 0.001	0.762
Group*time	25.433	2.000	12.717	6.436	0.003	0.192

**Table 8:** Comparison of VAS-QLV between and within groups before and after treatment.

**Group**	**VAS-QLV-pre**	**VAS-QLV-post**	**VAS-QLV-difference**	**Paired *t*-test**	***P***
A	7.5 ± 1.0	0.9 ± 0.9^*^^&^	6.6 ± 1.4^*^^&^	15.461	< 0.001
B	6.4 ± 2.2	2.7 ± 1.1	3.7 ± 2.4	4.959	0.001
C	6.6 ± 1.6	2.6 ± 1.4	4.0 ± 2.3	5.477	< 0.001
F	1.265	8.272	5.997		
P	0.299	0.002	0.007		

* Indicates a statistically significant difference from group C (*P* < 0.05).

& Indicates a statistically significant difference from group B (*P* < 0.05).

The above results of two-way ANOVA showed that there was no statistically significant difference between the overall groups (*P* > 0.05), there was a statistically significant difference between different time points (*P* < 0.05), and the interaction between time and group was a statistically significant difference (*P* < 0.05).

The before and after treatment scores on the VAS-QLV were 7.5 ± 1.0 and 0.9 ± 0.9 in Group A, 6.4 ± 2.2 and 2.7 ± 1.1 in Group B, and 6.6 ± 1.6 and 2.6 ± 1.4 in Group C, demonstrating a significant improvement in all groups (*P* < 0.001, *P* = 0.001, and *P* < 0.001, respectively). The comparison of treatment effect showed that VAS-QLV scores differed significantly between groups (*P* = 0.007). After training, the VAS-QLV in Group A decreased significantly compared with Group B and C (*P* < 0.05).

The results of VAS-A are shown in Tables 9, 10.

**Table 9:** The correlation between acceptance ratio and VAS-A severity.

	**VAS-A severity (** ***n*** **)**			**Acceptance**
	**Poor**	**General**	**Good**	**Ratio (%)**
Group A	0	3	7	70%
Group B	8	1	1	10%

VAS-A severity, scores of VAS-A severity.

**Table 10:** The analysis of correlation between age and VAS-A.

**Index**	**Statistic**	**A_Age**	**B_Age**
A_VAS-A	*R*	−0.356	–
	*P*	0.312	–
B_VAS-A	*r*	–	0.362
	*P*	–	0.304

In terms of acceptance, the VAS-A score was 7.6 ± 2.2 in Group A and 3.1 ± 2.8 in Group B (*P* = 0.004), The acceptance rate was 70% in group A and 10% in group B.

**Figure 1:**
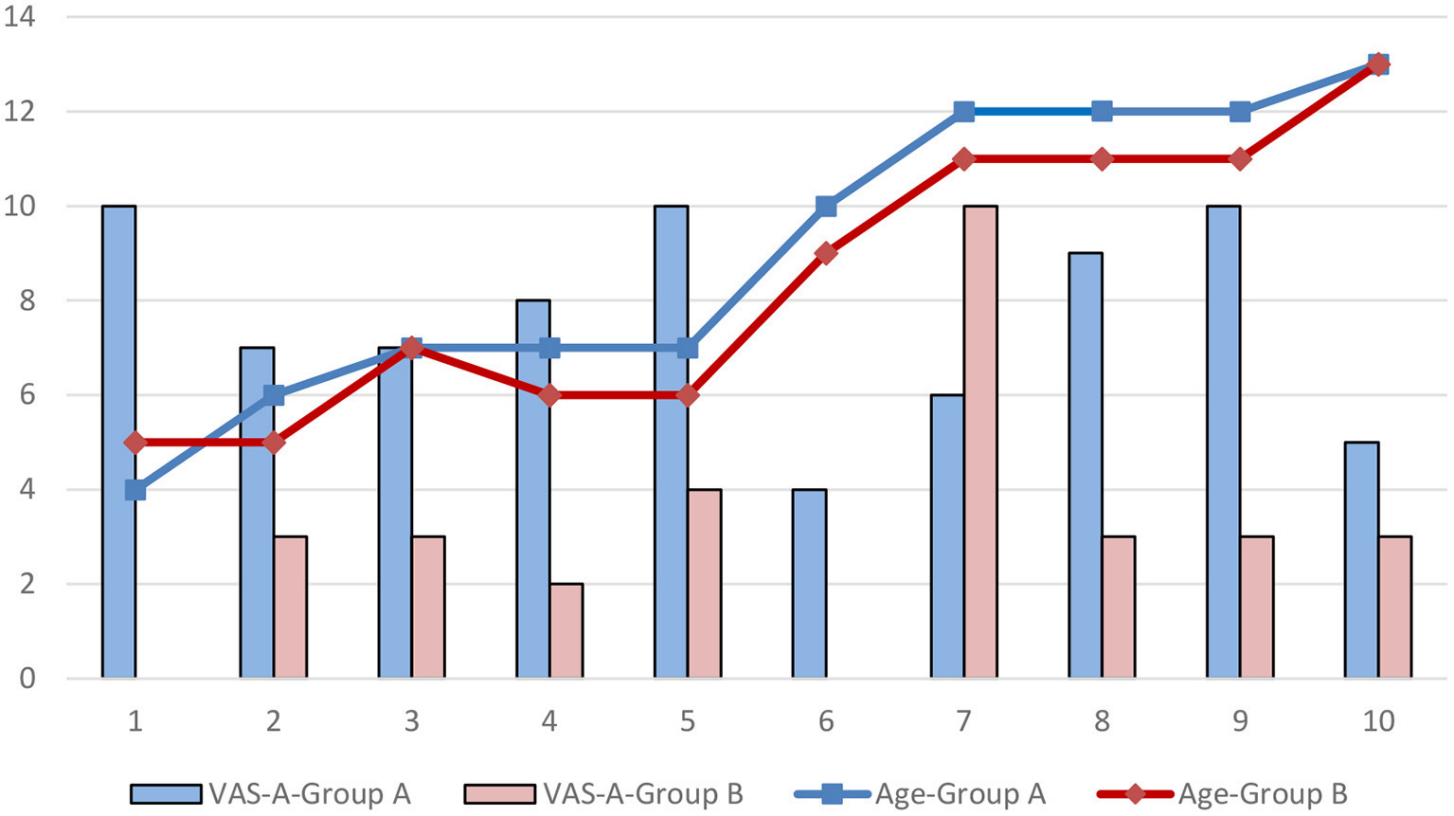
The relationship between age and VSA-A.

The relationship between the age and acceptance is shown in Figure 1. The results of Pearson correlation analysis showed that there was no significant correlation between age and VAS-A in either group A or group B (*P* > 0.05).

## 4. Discussion

Treatment measures are gradually improving with an increased focus on vertigo in children and improvements in diagnosis. The American physical therapy association neurology section published an Evidence-Based Clinical Practice Guideline of Vestibular Rehabilitation for Peripheral Vestibular Hypofunction. Clinicians should offer vestibular rehabilitation to patients with chronic unilateral vestibular hypofunction (Evidence quality: I; recommendation strength: strong) (4).

In addition to treating the symptoms, vestibular rehabilitation training is increasingly recommended as a treatment for children (16). However, its clinical application has limitations; the adopted training scheme and effect of training need to be further explored. The Cawthorne-Cooksey approach is one of several exercise programs that can be used in the treatment of unilateral vestibular hypofunction (7). However, many patients do not follow an exercise program for chronic dizziness. Many factors underlie this situation, such as the discomfort of emerging dizziness while exercising, an inability to devote time to exercise in one's daily life, a lack of enjoyment of exercise, and a lack of understanding regarding how exercises can correct dizziness (8). Because children's compliance and endurance are worse than adults, its applicability is worse in pediatric patients. therefore, the therapeutic purpose is not reached.

In this study, children aged 4–13 with RVC with unilateral peripheral vestibular dysfunction and normal balance function were treated with vestibular rehabilitation. According to the characteristics of moderate to severe impairment and repeated attacks of recurrent vertigo in children, we focus on the core of vestibular rehabilitation; adaptation training, especially VOR training (17), simplified the original training program, increased the frequency of reinforcement for a single training method, significantly improved applicability and completion, and enhanced the treatment efficiency. In order to enable children to better complete the training, Rine et al. (18) and Braswell and Rine (19) increased the difficulty level by 80% according to age. Nevertheless, for fixation stability training, children often fail to understand the meaning of shaking their heads quickly while keeping the target clear (16). The training procedure was filmed into a video that could guide the movements of the children. The video suggested the angle and speed of head turning and eye rotation, which was completed under the supervision of a guardian. The results of the study showed that Group A, the single-item intensive VOR adaptation training group, took 5 min each time to watch the guidance video, and the acceptance rate was 70%, which was significantly better than the 10% acceptance rate of Group B, the Cawthorne-Cooksey training group. The completion rate of Group A was 100% for a single training session per day and 30% for three training sessions per day, which was slightly insufficient, but the recovery rate of the caloric test and the marked effective rate were both 100%. This also suggests that we need to study the frequency and duration of training further to attain the best training routine. The training time of Group B was 20 min each time, the completion rate of a single session per day was 60%, the completion rate of three sessions per day was 10%, and the marked effective rate was 70%. During the return visit, it was found that the reason for not being able to complete the training well was the psychological resistance of children or parents caused by the long training time, thinking that training is a very complicated thing. This single-strength training is not only highly targeted but suitable for children and the coordination of short training is greatly improved.

In order to eliminate the influence of age and vertigo severity on the therapeutic effect, a block randomized controlled design was used for this study. The CP value in the caloric test represents the decreased value of the unilateral semicircular canal function, which is a quantitative assessment of the VOR function of the external semicircular canal (20, 21). The caloric test was conducted on all children, the results and CP values were recorded, and the training effect was objectively and quantitatively evaluated. For unilateral and bilateral vestibular dysfunction, DHI can sensitively reflect the effect of vestibular rehabilitation training (22). The DHI questionnaire was used to evaluate the subjective perception of vertigo symptoms in children (23, 24), and the VAS-QLV score was used to evaluate the impact of vertigo attacks on children's daily life and learning behavior. This confirmed the effectiveness of the training program from another dimension. In our study, the results showed that for such children, the VOR adaptation training, the Cawthorne-Cooksey training, and oral medication alone could improve the children's symptoms. The CP values decreased obviously after treatment of 1 month, which established statistically significant differences before and after treatment. Therefore, CP is significant from the perspective of treatment time. However, there are no significant differences between the interaction and post-intervention groups. Therefore, it can be assumed that spontaneous recovery after the onset of vertigo has occurred. Several different mechanisms are involved in the recovery of function following unilateral vestibular loss. These mechanisms include cellular recovery, spontaneous re-establishment of the tonic firing rate centrally, adaptation of residual vestibular function, the substitution of alternative strategies for the loss of vestibular function, and habituation of unpleasant sensations. Vestibular rehabilitation treatment should begin as early as possible, since there is evidence that early intervention with vestibular exercises facilitates a decrease in symptoms and improves gait stability compared with no exercises in patients with unilateral vestibular loss (25). In our study, intervention in 70% of cases occurred within 3 months. The spontaneous recovery may be due to vestibular compensation during the acute phase after the onset of vertigo. This may be related with the fact that the main outcome of DHI showed no interaction. Moreover, the suboutcome of VAS-QLV shows an interaction and a simple main effect after the intervention. Regarding this result, we believe that the intervention in this study is effective in improving subjective symptoms of vertigo. After training, the VAS-QLV in Group A decreased significantly compared with Group B and C. Compared with the classical Cawthorne-Cooksey program, the simple and targeted VOR adaptation training has the highest symptom alleviation efficiency, and the impact on life and learning behavior is improved more significantly, thus highlighting the advantages of the VOR adaptation training. Certainly, we will continue to follow up the patients to study the effect of each group's training regimen on the long-term vertigo alleviation efficacy in children.

The age applicability of this VOR adaptation training design is also high. The minimum age of the children in this study was 4 years, and the maximum was 13 years. Not only has it been confirmed that rehabilitation training for children with recurrent vertigo can provide a good effect, but also that rehabilitation training for young children has been conducted. Among them, the youngest was a 4-year-old child who completed the training in full accordance with the training approach, frequency, and sessions. Our study showed that the acceptance of rehabilitation training was not significantly related to age. It should be noted that pediatric patients still need to be trained under the guidance of the guardian, which also means that the guardian's compliance with treatment needs to be improved to complete the training and achieve its purpose.

## 5. Conclusions

For children with recurrent vertigo with unilateral vestibular dysfunction but normal balance function, single strengthening of VOR adaptation training can effectively improve vertigo symptoms, which is feasible and highly acceptable. However, the training frequency, duration, and long-term efficacy should be further discussed.

## Data availability statement

The original contributions presented in the study are included in the article/supplementary material, further inquiries can be directed to the corresponding authors.

## Ethics statement

The studies involving human participants were reviewed and approved by Medical Ethics Committee, Beijing Children's Hospital, Capital Medical University. Written informed consent to participate in this study was provided by the participants' legal guardian/next of kin. Written informed consent was obtained from the individual(s), and minor(s)' legal guardian/next of kin, for the publication of any potentially identifiable images or data included in this article.

## Author contributions

NM contributed to the conception of the study. NM, HL, BLiu, LZ, BLi, YY, WL, MC, JS, and XZ performed the experiment. NM and JZ contributed significantly to analysis and manuscript preparation. NM, HL, and JZ performed the data analyses and wrote the manuscript. NM, XN, and JZ helped perform the analysis with constructive discussions. All authors contributed to the article and approved the submitted version.
